# Ultrasound Imaging Analysis of the Lumbar Multifidus Muscle Echo Intensity: Intra-Rater and Inter-Rater Reliability of a Novice and an Experienced Rater

**DOI:** 10.3390/medicina57050512

**Published:** 2021-05-20

**Authors:** Maryse Fortin, Brent Rosenstein, Jerome Levesque, Neil Nandlall

**Affiliations:** 1 Department of Health, Kinesiology & Applied Physiology, Concordia University, Montreal, QC H4B 1R6, Canada; brent.rosenstein@concordia.ca (B.R.); levesque.jerome@hotmail.com (J.L.); n_nan@liveconcordia.onmicrosoft.com (N.N.); 2 PERFORM Centre, Concordia University, Montreal, QC H4B 1R6, Canada; 3 Centre de Recherche Interdisciplinaire en Réadaptation (CRIR), Constance Lethbridge Rehabilitation Centre, Montreal, QC H4B 1T3, Canada

**Keywords:** multifidus muscle, low back pain, ultrasound imaging, echo intensity, reliability

## Abstract

*Background and Objectives*: Ultrasound echo intensity (EI) of the lumbar multifidus muscle (LMM) could offer valuable insights regarding muscle quality in people with low back pain (LBP). However, whether the rater’s experience noticeably influences the reliability and precision of LMM EI measurements has not been established. The aims of this study were to investigate the intra-rater and inter-rater reliability of LMM EI measurements, and to compare the reliability and SEM between a novice and an experienced rater. *Materials and Methods*: Twenty athletes (10 females, 10 males) with a history of LBP were included in this study. Transverse ultrasound images of LMM were taken at L5 in prone. LMM EI measurements were obtained bilaterally by tracing the maximum ROI representing the LMM cross-sectional area (CSA), avoiding the inclusion of bone or surrounding fascia. All measurements were performed by two novice raters and an experienced researcher. Each measurement was acquired by each rater three times for each side on three different images, and the average was used in the analyses. Raters were blinded to each other’s measurements and the participant’s clinical information. Intra-class correlation coefficients (ICCs) were obtained to assess the intra-rater and inter-rater reliability. *Results*: The intra-rater ICC values for the LMM measurements for the experienced rater were excellent (ICC all > 0.997). The inter-rater reliability ICC values showed moderate to excellent reliability (0.614 to 0.994) and agreement between the novice raters and the experienced rater, except for Novice 1 for the right LMM, which revealed lower ICCs and a wider 95% CI. Intra-rater and inter-rater reliability results were similar when separately looking at the right and left side of the muscle and participant gender. *Conclusions*: Our findings support the clinical use of ultrasound imaging for the assessment of LMM EI in individuals with LBP.

## 1. Introduction

Biomechanical studies have highlighted the important role of the lumbar multifidus muscle (LMM) to provide arthrokinetic control of the vertebral segment, spinal stiffness [1,2], and proprioception of the lumbar spine [3]. Sufficient LMM function is essential to maintain the stability of the kinetic chain and generate forces to the lower and upper limbs [4]. Magnetic resonance imaging (MRI) and ultrasound imaging studies of athletes and non-athletes with LBP have reported morphological changes and functional deficits of the LMM, such as LMM atrophy [5,6,7,8,9], LMM cross-sectional area (CSA) asymmetry [5,7,10,11,12], increased fatty infiltration [13,14,15,16], and increased or decreased muscle activity [17,18,19]. Magnetic resonance imaging (MRI) remains the gold standard technique for muscle imaging, since the high resolution allows accurate assessment of muscle size and composition. However, ultrasound imaging is a more accessible and less expensive imaging technique that provides valuable information about muscle function [20,21,22]. LMM muscle CSA, thickness during submaximal contraction and at rest, and echo intensity (EI) in different postures can be measured with ultrasound to assess muscle size, function, and quality, respectively [23,24,25,26].

EI is measured using the ultrasound brightness scale via a gray scale analysis of the pixels in a region of interest (ROI). This measurement can be used as an indicator of muscle quality by estimating intramuscular fat and connective tissue [22,27,28,29] and muscle damage [30]. Young et al. [22] tested the reproducibility and inter-rater reliability of ultrasound EI in four different muscles and reported both high reproducibility and inter-rater reliability. The authors concluded that ultrasound is an accessible, cost-effective, reproducible imaging technique that is useful to examine skeletal muscle health [22]. This validation study also compared ultrasound EI to MRI, and reported moderate to strong correlations between muscle EI and percent intramuscular fat measured by MRI [22]; this finding is consistent with other studies comparing EI and muscle biopsy samples [31,32]. Similarly, EI of paravertebral muscles in people with chronic low back pain (LBP) is highly correlated with the amount of connective tissue and/or fatty infiltration assessed via biopsy [33]. The increased intramuscular fatty infiltration (e.g., decreased muscle quality) observed in people with chronic LBP [14,15,34] is theorized to increase the risk of injury and reduce overall muscle function [35,36,37]. Previous studies have also reported that muscle EI is correlated with percentage body fat [23,24,25,26], muscle strength and power [38,39,40], neuromuscular diseases [41,42], and lower cardiovascular performance [38,43,44]. Given these findings, as well as the relative novelty of assessing skeletal muscle EI using ultrasound and its potential clinical use, it is critical to further examine the reliability of this measurement.

Several factors can influence the reliability of EI muscle measurements, including the rater’s ultrasound experience, type of muscle, scanning site, and EI measurement method. Although some studies have examined the reliability of EI measurements of the LMM muscle [45,46,47] with high intra-rater reliability [45,47], none to our knowledge have compared the reliability and standard error of measurements (SEMs) between a novice rater and an experienced rater. Studies suggest that the variability in EI across different muscle groups is a result of the different distribution of fibrous tissue and the orientation of muscle fibers in each muscle group [29,48]. Young et al. [22] reported higher correlations when comparing percent intramuscular fat measured by MRI to corrected muscle EI of each muscle group than when comparing to all muscle groups. Young et al. [22] also found significant variability of muscle EI between two sites of each muscle group. Different EI measurement methods (e.g., size of ROI, imaging plane) influence the reliability of this measure [29,45]. Maximum ROI includes as much muscle as possible, avoiding bone and surrounding fascia [29], while other researchers use rectangular ROI [45]. Sarafraz et al. [45] reported superior intraclass correlation coefficients (ICCs) and SEM values when using maximum ROI in the transverse plane compared to maximum rectangular ROI in the longitudinal plane. Caresio et al. [29] reported moderate to high ICCs for within-session muscle EI depending on ROI size, with larger ROIs leading to higher reliability. Therefore, EI reliability is influenced by ROI size, and there still remains some controversy regarding the reliability of EI muscle measurement in the literature.

An adequate level of reliability is essential to use this ultrasound measure in a clinical setting. More specifically, whether the rater’s experience noticeably influences the reliability and precision of LMM ultrasound measurements has not been reported. Given that the LMM plays a key role in lumbopelvic control, the assessment of EI intra-rater and inter-rater reliability warrants further attention. Therefore, the primary aim of this study was to investigate the intra-rater and inter-rater reliability of LMM EI measurements, and to compare the reliability and SEM between a novice and an experienced rater.

## 2. Materials and Methods

### 2.1. Participants

Twenty young adults (10 females, 10 males) with a history of LBP from a larger study including soccer, hockey, football, and rugby university level varsity team players were randomly selected and included in the current study. The exclusion criteria included a previous history of severe trauma or spinal fracture, previous spinal surgery, observable spinal abnormalities, and pregnancy, as all of these could affect paraspinal muscle morphology and/or function. This study was approved by the central ethics committee of the Quebec Ministry of Health and Social Services (project #CCER-16-17-06, 21 July 2016). All participants provided informed consent acknowledging that their data would be used for research purposes.

### 2.2. Procedures

A self-administered questionnaire was completed to collect information regarding participants’ demographics and history of LBP. LBP was defined as pain localized between T12 and the gluteal fold [49]. Participants were asked to answer yes or no to the presence of LBP during the past four weeks (pre-season) or three months (off season) prior to the assessment. Players who answered yes to the presence of LBP completed a Numerical Pain Rating Scale (NPRS) to assess average LBP intensity. Participants were also asked about pain location (e.g., center, right side, left side) and pain duration (in months) at both time points. All 20 players in the current study reported LBP in the past four weeks and/or three months prior to measurement.

### 2.3. Ultrasound

Ultrasound B-mode images of the LMM were captured using a LOGIQ e ultrasound machine (GE Healthcare, Milwaukee, WI, USA) with a 5 MHz curvilinear transducer. The imaging parameters were kept consistent in all acquisitions (frequency: 5 MHz, gain: 60, depth: 8.0 cm).

Participants were placed on a therapy table in a prone position with a pillow under their abdomen to minimize lumbar lordosis [50]. Participants were instructed to relax the paraspinal musculature while the images were obtained. Prior to imaging, the L5 spinous process was palpated and labeled on the skin with a pen. Acoustic coupling gel was administered on the skin, and the ultrasound transducer was positioned longitudinally along the midline of the lumbar spine to detect the location of L5. The transducer was then rotated and positioned transversally over the L5 spinous process for imaging. Transverse images of the LMM at L5 were taken bilaterally, except for larger muscles, where the right and left sides were imaged separately. A total of three images were obtained bilaterally for the right and left LMM.

### 2.4. Images Assessment

**Figure 1:**
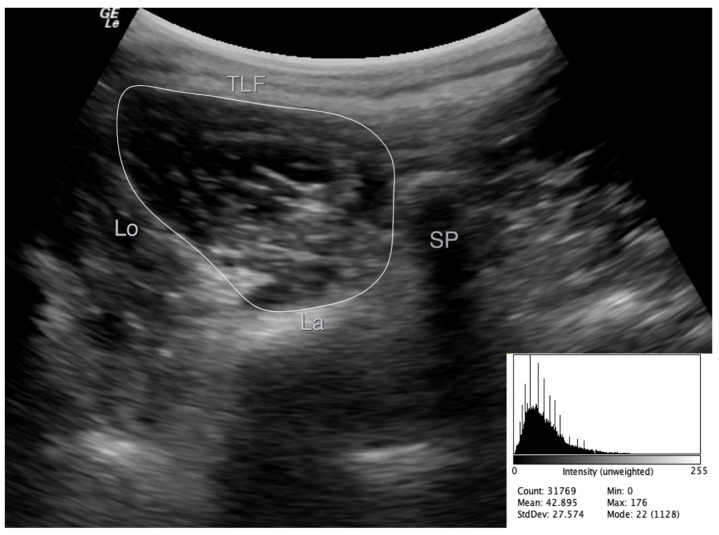
Example of an echo intensity measurement of the left multifidus muscle at L5 from the transverse image and important landmarks used to define the muscle borders. TFL: thoracolumbar fascia, SP: spinous process, La: echogenic laminae, Lo: longissimus muscle.

The images were stored and analyzed offline. LMM EI was measured using grayscale analysis imaging via ImageJ software (National Institute of Health, Bethesda, MD, USA, Version 1.49). EI was determined by tracing the maximum ROI representing the LMM cross-sectional area (CSA), avoiding the inclusion of bone or surrounding fascia (Figure 1). EI was then defined as the mean level of gray within the ROI using the grayscale histogram function (e.g., pixels expressed as a value between 0 = black and 255 = white) [27], where enhanced EI is indicative of a greater amount of intramuscular fat and connective tissue. The EI measurements were acquired three times for each side on three different images, and the average was used in the analyses.

All muscle measurements were performed by two novice raters (athletic therapy students; Novice 1, N.N. and Novice 2, J.L.) and an experienced researcher (M.F.) with over 10 years of experience in paraspinal imaging analysis. In preparation for this study, the novice raters received training from the experienced rater regarding the anatomy, ultrasound imaging assessment, and how to acquire the LMM CSA and EI measurements. For practice purposes, the novice raters analyzed a sample of five participant images before the start of the measurement study, which were then evaluated and approved by the experienced rater. Then, each rater obtained the muscle measurement three times (on three differing images) on each side, while blinded to each other’s measurements and the participant’s clinical information. Measurements were obtained once by both novice raters and twice by the experienced rater (with seven days between each time the same image was measured).

### 2.5. Statistical Analysis

Means and standard deviations were calculated for participants’ characteristics and the LMM measurements. The intra-rater reliability for the experienced rater (M.F.), the inter-rater reliability between Novice 1 (N.N.) and the experienced rater (M.F.), and between Novice 2 (J.L.) and the experienced rater (M.F.) were determined by computing the intraclass correlation coefficient (ICC) for the LMM EI measurement. For the intra-rater reliability, the ICC was calculated using a two-way mixed model, average measurement, and absolute agreement. For the inter-rater reliability, the ICC was calculated using a two-way random effects model, average measurement, and absolute agreement. The reliabilities for the right and left LMM were assessed separately. The reliabilities for the male and female participants were assessed as well. The ICCs were interpreted using the following classification, as suggested by Portney and Watkins [51]: less than 0.5 indicates poor, 0.50–0.74 indicates moderate, 0.75–0.90 indicates good, and greater than 0.90 indicates excellent. The SEM was also calculated to provide an estimate of the expected error related to each muscle measurement. Statistical analysis was performed with the IBM SPSS Statistics version 25.0 (IBM Corp., Armonk, NY, USA).

## 3. Results

### 3.1. Participants

The mean ± SD age, height, and weight was 21.2 ± 1.3 years, 173.4 ± 10.1 cm, and 75.0 ± 12.3 kg, respectively. A total of 90% (*n* = 18) reported LBP during the pre-season (past four weeks) and 95% (*n* = 19) during the off season (past three months), with a severity of 3.5 ± 1.2 and 4.3 ± 2.0 on the NPRS, respectively. Descriptive data (mean and standard deviation) of participants’ baseline characteristics are presented in Table 1.

### 3.2. Intra-Rater Reliability

The intra-rater reliability results for LMM measurements of the right and left side for the experienced rater are presented in Table 2. The ICCs indicated excellent intra-rater reliability for all participants, and ranged from 0.997 to 1.000. The SEM values for all participants were relatively small, and ranged between 0.443 and 0.511. The results were similar when separately looking at the right and left side of the muscle, as well as participant gender.

### 3.3. Inter-Rater Reliability

The inter-rater reliability results for LMM measurements of the right and left side between the novice raters and the experienced rater are presented in Table 3. The ICCs indicated moderate to excellent inter-rater reliability between Novice 1 and the experienced rater for all participants, and ranged from 0.614 to 0.994. The SEM values for all participants were relatively small, and ranged between 1.999 and 3.308. The results were similar when separately looking at the right and left side of the muscle, as well as participant gender. Notably, the inter-rater reliability between Novice 1 and the experienced rater for the right LMM showed lower ICCs, a wider 95% CI, and larger SEM values. The ICCs indicated excellent inter-rater reliability between Novice 2 and the experienced rater for all participants, and ranged from 0.897 to 0.997. The SEM values for all participants were relatively small, and ranged between 1.432 and 2.257. The results were similar when separately looking at the right and left side of the muscle, as well as participant gender.

## 4. Discussion

The purpose of this study was to investigate the intra-rater and inter-rater reliability of LMM EI measurements using transverse ultrasound images and maximum ROI in 20 participants with a history of LBP, and to compare the reliability and SEM between a novice and an experienced rater. The intra-rater ICC values for the LMM measurements for the experienced rater were all greater than 0.997, indicating excellent reliability. The inter-rater reliability ICC values showed moderate to excellent reliability and agreement between the novice raters and the experienced rater, except for Novice 1 for the right LMM, which revealed lower ICCs and a wider 95% CI. Overall, the intra-rater and inter-rater reliability results were similar when separately looking at the right and left side of the muscle, as well as participant gender.

### 4.1. Intra-Rater Reliability

Our findings are similar to another study examining the intra-rater reliability of ultrasound LMM EI measurements. Sarafraz et al. [45] investigated within-day intra-rater reliability of several muscles, including the LMM, in 15 participants with a complaint of LBP and 15 healthy controls. The authors measured EI using two orientations, transverse and longitudinal, and captured three ultrasound images in each orientation for each muscle side. In addition, the authors used two different ROI methods: (1) maximum ROI, which includes as much muscle as possible (e.g., the CSA of the LMM in the transverse plane), and (2) a maximum rectangular ROI (longitudinal); the average of the three measurements was used in the analysis. In participants with LBP, using the transverse images and maximum ROI, the authors reported similar ICCs to our study. Their reliability results, however, differed depending on the ROI method and image orientation. Sarafraz et al. [45] reported better reliability with higher ICCs and lower SEM values when using max ROI (e.g., representing the CSA of the LMM) and transverse images in all participants. Similarly, Caresio et al. [29] reported moderate to high ICCs for within-session gastrocnemius and tibialis anterior muscle EI depending on ROI size, with larger ROIs correlating with higher reliability. Therefore, EI reliability is influenced by the ROI size and image orientation, and there still remains some controversy regarding the reliability of LMM EI measurement in the literature.

Yamamoto and Miyazaki [47] investigated between-day (ICCs: right LMM = 0.898 (0.662–0.970), left LMM = 0.775 (0.252–0.934)) and within-day (ICCs: right LMM = 0.981 (0.936–0.994), left LMM = 0.981 (0.935–0.994)) intra-rater reliability of the LMM in 12 healthy participants. Measurements were obtained twice for each side of the LMM on the same day, and on different days using the same maximum ROI definition as our study. Additionally, Resende et al. [46] investigated between-day and within-day intra-rater reliability of the superficial and deep layers of the LMM (e.g., using an ROI box of 1 cm^2^) in 31 volunteers (e.g., did not report LBP for the past three months) using both transverse and longitudinal images. Interestingly, the authors reported greater ICC values when using bilateral longitudinal images as opposed to transverse images. Overall, our study had higher intra-rater ICCs and lower SEM values than Resende et al. [46]. However, our ROI method included the entire LMM muscle, and made no distinction between the superficial and deep LMM muscle fibers. 

Differences between our findings and the previous studies [46,47] could be explained due to methodological differences. In the current study, intra-rater reliability was assessed by repeating the measures on the same ultrasound images seven days apart. Resende et al. [46] and Yamamoto and Miyazaki [47] assessed between-day intra-rater reliability, repeating all procedures on participants during a second visit, which inevitably increased the measurement error. In addition, our study used the average of three measurements from a transverse ultrasound image, while Yamamoto and Miyazaki [47] used the average of two measurements from longitudinal images. As mentioned previously, using different image orientations appears to influence the reliability of EI measurements. Koppenhaver et al. [52] investigated improvements in precision in muscle thickness measurements of the LMM using ultrasound imaging in 30 participants with LBP. The authors reported that, compared to one measurement, the SEM decreased by almost 25% when using an average of two measurements and nearly 50% when using the average of three measurements. The ICC values (95% CI) of the LMM when using a single measurement and the mean of two and three measures also increased from 0.88 (0.76–0.94), 0.94 (0.87–0.97), and 0.96 (0.92–0.98), respectively. The authors stated that measurement precision is optimized by averaging three measures of the LMM. However, averaging more than three measurements led to little or no further improvements in precision [52]. Therefore, it is possible that using an average of three measurements in our study led to a higher reliability and lower SEM compared to averaging two measurements. Furthermore, a study by Resende et al. [46] also investigated the reliability EI measurement of the deep portion of the LMM muscle as opposed to the entire LMM, as measured in the current study. Making a distinction between the deep and superficial LMM fibers may affect the reliability due to the arbitrary position of the ROI, and the thickness of the LMM will also vary according to individual anthropometric differences. Interestingly, the authors reported higher EI values for the superficial layer than the deep layer. The authors explained that, due to attenuation in the upper parts of the LMM, less energy reaches the deeper layer, making the superficial layer more distinguishable and leading to less variability [46]. Imaging studies have also shown that fatty deposits within the LMM are not homogeneous, and primarily occur in the medial part and deepest layer of the muscle [35,53]. This likely explains why they reported higher ICC values and lower SEM values for the superficial layer compared to the deeper layer.

There are several other factors that can influence the reliability of EI measurements, including the rater’s level of experience, type of muscle, and imaging site. Although some studies have examined the reliability of EI measurements of the LMM muscle [45,46,47] with high intra-rater reliability [45,47], none to our knowledge have compared the reliability and SEMs between a novice rater and an experienced rater. Studies suggest that the variability in EI across different muscle groups is a result of the different distribution of fibrous tissue and the orientation of muscle fibers in each muscle group [29,48]. Young et al. [22] reported higher correlations when comparing percent intramuscular fat measured by MRI to corrected muscle EI of each muscle group than when comparing to all muscle groups. The authors also found significant variability of muscle EI between two sites of each muscle group and percent intramuscular fat between three different MRI slices. This shows the importance of keeping scanning sites consistent, and is in line with the findings of Scholten et al. [54], which emphasized the necessity of measuring the precise muscle location to acquire comparable and reliable EI results across individuals.

An additional factor that can influence the reliability of EI measurements is the participant’s gender. To our knowledge, no other studies have separately analyzed the reliability of LMM EI measurements by the participant’s gender. However, it is important to note that Fortin et al. [23] and Nandlall et al. [24] showed significantly greater LMM EI values in female young adults than in male young adults when assessing the muscle at rest in a prone position. They concluded that this finding was likely due to females having a greater amount of LMM fatty infiltration/connective tissue due to a naturally higher percentage of body fat, which supports previous findings [14,15,34,55]. A strong correlation was reported between total percent body fat and LMM EI for both male and females [22,23]. Although the current study revealed that LMM EI reliability indices were similar between the male and female participants, this needs to be replicated in the older population.

### 4.2. Inter-Rater Reliability

Overall, both of the inter-rater ICCs showed moderate to excellent reliability between the novice raters and the experienced rater, except for Novice 1 for the right LMM, which revealed lower ICCs and a wider 95% CI, showing a lack of agreement between the two raters. The results were similar when separately looking at the right and left side of the muscle, as well as participant gender. In addition, our results for both of the novice raters, specifically the right LMM for Novice 1, showed larger SEM values compared to the experienced rater. We are not aware of any other studies that have examined the effect of the rater’s level of experience in acquiring LMM EI measurements. However, our findings are in accordance with other studies that have examined EI measures in other muscle groups [56,57,58].

Ishida et al. [56] investigated the inter-rater reliability of rectus femoris muscle EI measurements in 14 healthy male participants. The authors reported an inter-rater ICC of 0.95 and an SEM of 0.9 between a rater with six years of ultrasound experience and a rater with one year of experience. Rabello et al. [57] investigated the inter-rater reliability of rectus femoris muscle EI measurements in 32 healthy participants (50% female) between a rater with four years of ultrasound experience and a rater with one year of experience. Ultrasound images were obtained at 50% of the rectus femoris belly length and at 70% of the rectus femoris belly length. At 50% of the rectus femoris belly length, the authors reported inter-rater ICCs of 0.89 (0.82–0.94) and an SEM of 3.95 between the two different experienced raters. At 70% of the rectus femoris belly length, the authors reported inter-rater ICCs of 0.90 (0.82–0.94) and an SEM of 3.87 between the two raters. Therefore, the inter-rater ICCs between the two raters were excellent at each belly length. Lee et al. [58] investigated the inter-rater reliability of abductor pollicis brevis and abductor digiti minimi muscle EI measurements in 20 age-matched controls and 20 patients with carpal tunnel syndrome. The measurements were performed by an experienced rater and a novice rater, but the level of experience for each rater was not mentioned. The authors reported an inter-rater ICC of 0.897 and 0.837 for the abductor pollicis brevis and abductor digiti minimi muscle, respectively. Accordingly, it appears that even with minimal experience or training by the rater, the reliability of EI muscle measurements are good to excellent [56,57,58]. These findings are similar to our study, which showed good to excellent reliability between the novice raters and the experienced rater, except for Novice 1 for the right LMM, which revealed lower ICCs and a wider 95% CI. Since the novice raters in our study had minimal training by only analyzing a sample of five participants prior to the study, one could expect that more training would have improved the agreement between the two raters.

### 4.3. Strengths and Limitations

A limitation of this study is the relatively small sample size, which included young and active participants. The reliability of the EI measurements should still be investigated in older and more sedentary individuals, since this factor affects the quality of the ultrasound images. Furthermore, we had no control group. However, our study did investigate individuals with LBP, in which EI was shown to correlate with connective tissue and/or fatty infiltration within muscle tissue in past studies. In addition, to our knowledge, this is the first study to compare the reliability of LMM EI measurements and SEM between a novice and an experienced rater.

## 5. Conclusions

Our findings suggest that EI LMM measurements using a maximum ROI in the transverse plane have excellent intra-rater reliability (experienced rater) and moderate to excellent reliability between novice and experienced raters. The reliability was also comparable between muscle side (right vs. left) and participant gender. While our results suggest that good reliability was achieved with minimal training from the novice raters, other factors can influence EI reliability measurements, including the type of muscle, imaging site, and ROI size. Our findings support the clinical use of ultrasound imaging for the assessment of LMM EI in individuals with LBP. This measure can offer valuable insights regarding muscle quality of the LMM, which plays a key role in lumbopelvic control.

## Figures and Tables

**Table 1:** Participants’ characteristics.

	All (*n* = 20)	Female (*n* = 10)	Male (*n* = 10)
Age (yr)	21.2 ± 1.3	21.3 ± 1.3	21.0 ± 1.3
Height (cm)	173.4 ± 10.1	166.5 ± 9.1	180.3 ± 5.3
Weight (kg)	75.0 ± 12.3	68.0 ± 11.8	81.9 ± 8.6
BMI (kg/m^2^)	24.8 ± 2.6	24.4 ± 2.4	25.3 ± 2.9
Sport, *n* (%)			
Hockey	11 (55)	5 (50)	6 (60)
Soccer	5 (25)	3 (30)	2 (20)
Rugby	4 (20)	2 (20)	2 (20)
Competitive level (yr) ^a^	9.3 ± 4.3	8.6 ± 4.6	10.0 ± 4.0
University level (yr)	2.5 ± 2.2	2.1 ± 1.4	2.8 ± 2.8
LBP four weeks prior, *n* (%)	18 (90)	9 (90)	9 (90)
LBP three months prior, *n* (%)	19 (95)	10 (100)	9 (90)
LBP last competitive year, *n* (%)	12 (60)	6 (60)	6 (60)
LBP location four weeks prior, *n* (%) ^b^			
Centered	5 (28)	2 (22)	3 (33)
Bilateral	6 (33)	3 (33)	3 (33)
Unilateral	7 (39)	4 (44)	3 (33)
LBP location three months prior, *n* (%) ^a^			
Centered	7 (37)	4 (40)	3 (33)
Bilateral	8 (42)	3 (30)	5 (56)
Unilateral	4 (21)	3 (30)	1 (11)
NPRS LBP (0–10) four weeks prior ^b^	3.5 ± 1.2	3.5 ± 1.4	3.4 ± 1.1
NPRS LBP (0–10) three months prior ^a^	4.3 ± 2.0	3.8 ± 2.3	4.8 ± 1.5

Values are presented as means ± SDs unless otherwise specified. BMI: body mass index; LBP: lower back pain; NPRS: Numerical Pain Rating Scale. ^a^—One missing piece of data from the male group. ^b^—One missing piece of data from each male and female group.

**Table 2:** Intra-rater reliability of LMM measurements by the experienced rater.

Participants and Side	ICC (95% CI)	SEM
All (*n* = 20)		
Right	0.999 (0.998–1.000)	0.511
Left	0.999 (0.997–0.999)	0.443
Female (*n* = 10)		
Right	0.999 (0.996–1.000)	0.466
Left	0.997 (0.986–0.999)	0.592
Male (*n* = 10)		
Right	0.997 (0.989–0.999)	0.608
Left	0.999 (0.998–1.000)	0.424

ICC: intraclass correlation coefficient; CI: confidence interval; SEM: standard error of measurement.

**Table 3:** Inter-rater reliability of LMM measurements between each novice and the experienced rater.

Novice 1 (N.N.) and Experienced Rater		Novice 2 (J.L.) and Experienced Rater			
Participants and Side	ICC (95% CI)	SEM	Participants and Side	ICC (95% CI)	SEM
All (*n* = 17) ^a^			All (*n* = 20)		
Right	0.963 (0.614–0.991)	3.308	Right	0.992 (0.974–0.997)	1.432
Left	0.982 (0.914–0.994)	1.999	Left	0.972 (0.897–0.990)	2.257
Female (*n* = 8)			Female (*n* = 10)		
Right	0.977 (0.372–0.996)	2.351	Right	0.993 (0.963–0.998)	1.237
Left	0.972 (0.661–0.995)	1.854	Left	0.976 (0.895–0.994)	1.707
Male (*n* = 9)			Male (*n* = 10)		
Right	0.898 (0.241–0.980)	3.954	Right	0.978 (0.913–0.994)	1.614
Left	0.976 (0.890–0.995)	2.246	Left	0.953 (0.759–0.989)	2.701

^a^—One missing piece of data from the male group and two missing data from the female group; ICC: intraclass correlation coefficient; CI: confidence interval; SEM: standard error of measurement.

## Data Availability

The data presented in this study are available on request from the corresponding author.
